# Sequential analysis of transcript expression patterns improves survival prediction in multiple cancers

**DOI:** 10.1186/s12885-020-06756-x

**Published:** 2020-04-07

**Authors:** Jordan Mandel, Raghunandan Avula, Edward V. Prochownik

**Affiliations:** 1 The Division of Hematology/Oncology, Children’s Hospital of Pittsburgh of UPMC, Rangos Research Center, Room, 5124, 4401 Penn Ave, Pittsburgh, PA 15224 USA; 2 The Hillman Cancer Center of The University of Pittsburgh Medical Center, UPMC, 5150 Centre Ave, Pittsburgh, PA 15232 USA; 3 The Pittsburgh Liver Research Center, S414 Biomedical Science Tower, 200 Lothrop Street, Pittsburgh, USA; 4 The Department of Microbiology and Molecular Genetics, 450 Technology Dr, Pittsburgh, PA 15219 USA

**Keywords:** Transcriptional profiling, Signal transduction, Tumor metabolism, Dimensionality reduction, T-SNE

## Abstract

**Background:**

Long-term survival in numerous cancers often correlates with specific whole transcriptome profiles or the expression patterns of smaller numbers of transcripts. In some instances, these are better predictors of survival than are standard classification methods such as clinical stage or hormone receptor status in breast cancer. Here, we have used the method of “t-distributed stochastic neighbor embedding” (t-SNE) to show that, collectively, the expression patterns of small numbers of functionally-related transcripts from fifteen cancer pathways correlate with long-term survival in the vast majority of tumor types from The Cancer Genome Atlas (TCGA). We then ask whether the sequential application of t-SNE using the transcripts from a second pathway improves predictive capability or whether t-SNE can be used to refine the initial predictive power of whole transcriptome profiling.

**Methods:**

RNAseq data from 10,227 tumors in TCGA were previously analyzed using *t-*SNE-based clustering of 362 transcripts comprising 15 distinct cancer-related pathways. After showing that certain clusters were associated with differential survival, each relevant cluster was re-analyzed by t-SNE with a second pathway’s transcripts. Alternatively, groups with differential survival identified by whole transcriptome profiling were subject to a second, t-SNE-based analysis.

**Results:**

Sequential analyses employing either t-SNE➔t-SNE or whole transcriptome profiling➔t-SNE analyses were in many cases superior to either individual method at predicting long-term survival. We developed a dynamic and intuitive R Shiny web application to explore the t-SNE based transcriptome clustering and survival analysis across all TCGA cancers and all 15 cancer-related pathways in this analysis. This application provides a simple interface to select specific t-SNE clusters and analyze survival predictability using both individual or sequential approaches. The user can recreate the relationships described in this analysis and further explore many different cancer, pathway, and cluster combinations. Non-R users can access the application on the web at https://chpupsom19.shinyapps.io/Survival_Analysis_tsne_umap_TCGA. The application, R scripts performing survival analysis, and t-SNE clustering results of TCGA expression data can be accessed on GitHub enabling users to download and run the application locally with ease (https://github.com/RavulaPitt/Sequential-t-SNE/).

**Conclusions:**

The long-term survival of patients correlated with expression patterns of 362 transcripts from 15 cancer-related pathways. In numerous cases, however, survival could be further improved when the cohorts were re-analyzed using iterative t-SNE clustering or when t-SNE clustering was applied to cohorts initially segregated by whole transcriptome-based hierarchical clustering.

## Background

Tumor-associated DNA copy number variations, gene fusions and gene expression differences have long been used to diagnose certain types of cancers, to forecast survival and to determine the necessity for or response to adjuvant chemotherapy, with some of these tests now being routinely employed as standard of care [1–6]. For example, the analysis of tumors from women with Stage I or II breast cancer using a 70 gene expression signature has permitted a highly accurate determination of whether these individuals are likely to benefit from post-surgical adjuvant chemotherapy [6–8]. A shortcoming of such tests however is their applicability to only certain cancer types or even certain stages [4, 6]. Tests that rely on the expression of a common set of genes with predictive value across multiple cancer types have yet to be developed or implemented even though they could potentially reduce test complexity and cost.

In murine models of hepatoblastoma and hepatocellular carcinoma (HCC), we have previously observed that most transcripts encoding the 80 proteins comprising the 40S and 60S ribosomal subunits were significantly increased relative to those in normal livers [9, 10]. Because these increases were not uniform, the overall transcript expression patterns were altered as well. To determine whether this latter feature might be of prognostic value in human cancers, we used t-SNE [11] to profile the ribosomal protein transcript (RPT) expression patterns of 377 human HCC patients and 55 matched liver tissues whose transcriptomes had been deposited in The Cancer Genome Atlas (TCGA). This approach identified three distinct RPT “t-SNE clusters” in HCCs that were associated with significantly different long-term survival differences. RPT t-SNE pattern differences were also associated with survival differences in six other cancer types [9]. We subsequently used this same approach to classify the expression patterns of 25 transcripts encoding cholesterol biosynthesis enzymes and 37 mitochondrial fatty acid β-oxidation (FAO) enzyme transcripts into small numbers of t-SNE clusters [12]. Cholesterol biosynthesis-associated transcript clusters correlated with survival in eight cancer types and those for FAO correlated with survival in seven cancer types. The patterns of transcripts and the number of t-SNE clusters for each pathway and for ribosomal proteins were distinct for each cancer type. Collectively, these three pathways’ 142 component transcripts, predicted survival in 17 of the 34 different cancer types in TCGA, comprising 70.2% of all tumors. In six cancers, two pathways were predictive of survival.

Following this, we performed a more comprehensive TCGA-wide study on 220 transcripts from an additional twelve pathways, each comprised of 6–30 mRNAs [13]. While neither unique to cancer nor comprehensive in their scope, these pathways were selected because of their unequivocal roles in tumor cell growth, signaling and metabolism and included, among others, those comprising the cell cycle, Hippo, TGF-β and PI3 kinase signaling and several metabolic pathways [14]. As a group, t-SNE clusters of these transcripts were predictive of survival in 30 of 34 cancer types comprising 91.4% of all tumors. They were not predictive of survival in diffuse large B-cell lymphoma, lung squamous cell carcinoma cancer, pheochromocytoma+paraganglioneuroma and testicular germ cell tumor even when combined with the previously tested RPT, cholesterol biosynthesis and FAO pathway transcripts. Excluding the above four cancers, long-term survival in the remaining 30 were associated with an average of 3.6 pathways/tumor type (range one-nine). In some cases, t-SNE analysis could be used to further refine survival prediction among patients who had been previously well-stratified by such classical criteria as hormone receptor status in the case of breast cancer or by clinical staging in bladder cancer and head and neck cancer.

The above findings raised the question of whether the sequential analysis of tumors with transcripts from two different pathways might afford a more accurate and/or sensitive means of evaluating survival than is attainable with only a single such analysis. A related question is whether t-SNE analysis could also be applied to patient cohorts with distinct survival differences initially identified based on whole transcriptome profiling [13].

In the current work, we have utilized the above approaches, which we term “sequential t-SNE profiling” and “sequential hierarchical clustering/t-SNE profiling” to further improve long-term survival prediction of individual patient cohorts. Those tumors initially segregating into favorable or unfavorable long-term survival groups based on an initial assessment by t-SNE or whole transcriptome profiling are shown to be further divisible into groups that differ significantly in their long-term survival when a second round of analysis is performed using t-SNE profiling. These sequential approaches afford further refinements in long-term survival stratification.

## Methods

### Tumor selection

RNAseq data were obtained from the 10,227 newly diagnosed and previously untreated cancers of all stages, comprising 34 distinct types, currently maintained in TCGA along with pertinent clinical and demographic data. FPKM-UQ were obtained from the TCGA GDC PANCAN dataset and through the University of California Santa Cruz UCSC Xenabrowser as previously described [9, 12, 13]. Expression values were initially stored as the log_2_ of the incremented-by-one FPKM-UQ value. The inverse of this transformation was applied to the values to obtain the true FPKM-UQ values.

### Transcript analyses

Transcript selection, normalization and t-SNE dimensionality reduction were conducted as described previously [13]. Briefly, clinical data and transcript abundances normalized to FPKM-UQ were downloaded from the GDC PANCAN dataset and accessed via the UCSC Xenabrowser (https://sxena.ucsc.edu). For each pathway, transcript abundances were normalized to 1 across each sample, and projected onto a unit hypersphere. t-SNE dimensionality reduction was performed using Tensorboard v. 1.0 [15] and clustering was performed using Gaussian mixture models in MATLAB. Hierarchically clustered heatmaps were obtained from tcga.ngchm.net. Survival analyses were performed using the MatSurv (Anders) package for MATLAB (The Mathworks Corp. Nattick MA). Tests for non-random associations of membership between clusters were Fisher’s Exact Tests conducted in Graphad Prism 7 (GraphPad Software, San Diego CA). Interactive application was developed using R, a language and environment for statistical computing and the package “shiny” (R Foundation for Statistical Computing, Vienna, Austria).

### Application implementation

The application was developed using R Shiny and employs a combination of pre-generated data and dynamically created survival plots. t-SNE profiling was preformed previously as described previously for each cancer + pathway combination [9, 12, 13]. The R package *plotly* was used to generate 3D plots of t-SNE clustering, *survminer* and *survival* were used to generate survival curves using this t-SNE profiling, and *complexHeatmap* was used to pre-generate heatmap objects with annotations using TCGA expression data. Pre-generating these heatmap objects enables quick loading of large expression data and improved usability. Shiny reactive elements and conditional input panels were used to create an intuitive application that reveals input buttons as users make subsequent selections and provides instructions as the user navigates through the application (R Foundation for Statistical Computing, Vienna, Austria).

### Application design

The user is first asked to select the cancer transcriptome data from TCGA requiring analysis. They then choose a pathway for analysis, can explore the t-SNE clustering of the previously chosen cancer in the interactive 3D plots in *Tab. 1* and can view the survival differences among these clusters in *Tab. 2*. The user may then select a second pathway, which displays the clustering and survival differences among the previously analyzed t-SNE clusters in Tabs 1 and 2 respectively. Using the individual pathway survival curves in Tab. 2, the user can then select which cluster or clusters they wish to analyze using the “sequential t-SNE profiling approach”. *Tab. 3* displays the survival curves generated dynamically using this approach from the selected clusters and shows a *p*-value of the significance survival differences. *Tab. 5* shows a whole transcriptome profiling heatmap for the selected cancer that is annotated with clusters from t-SNE profiling using the first selected pathway and dendrogram groups from hierarchical clustering on TCGA expression data obtained from tcga.ngchm.net. The heatmap for this cancer can also be viewed directly on the NC-GHM viewer using the button at the bottom of *Tab. 5*. From this heatmap, one can select a dendrogram group or groups upon which to perform “sequential hierarchical clustering t-SNE profiling” and view the resulting survival curve in *Tab. 4*. This application reacts dynamically to changes in cluster selection such that choosing a new cancer or pathway resets the application to an earlier step in the sequence of steps described above. This interface provides a simple, user-directed exploration of the numerous combinations of pathways, clusters, and approaches of sequential analysis.

## Results

### Sequential t-SNE profiling

**Fig. 1:**
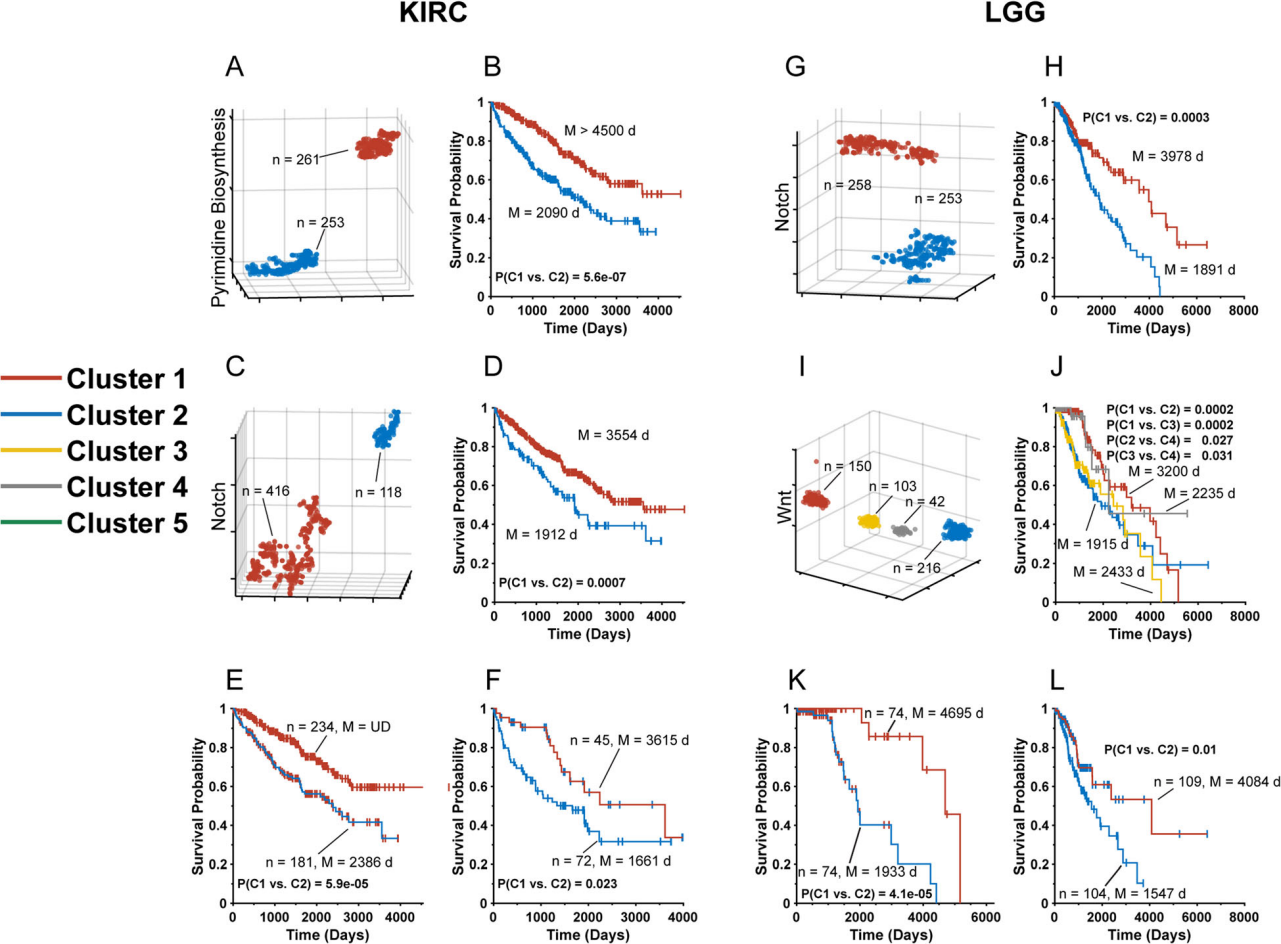
Sequential t-SNE analysis of KIRC and LGG. **a**. t-SNE-generated patterns of KIRC tumor Pyrimidine Biosynthesis Pathway transcripts showing two distinct clusters. n = number of tumors in each group **b**. Kaplan-Meier survival curves of the patient groups corresponding to the tumor clusters in A. M = median survival (in days) of each of the groups. **c**. t-SNE patterns of Notch Pathway transcripts. **d**. Kaplan-Meier survival curves of the patient groups corresponding to the tumor clusters in C. **e**. Kaplan-Meier survival of the favorable survival Notch Pathway cluster from D after t-SNE analysis using transcripts from the Pyrimidine Biosynthesis Pathway. Two t-SNE clusters similar to those depicted in A were observed (not shown). **f**. Kaplan-Meier survival of the unfavorable survival Notch Pathway cluster from D. **g**. t-SNE-generated patterns of LGG Notch Pathway transcripts showing two distinct clusters. **h**. Kaplan-Meier survival curves of each of the patient groups corresponding to the tumor clusters in (G). **i**. t-SNE-generated patterns of LGG Wnt Pathway transcripts showing four distinct clusters. **j**. Kaplan-Meier survival curves of each of the patient groups corresponding to the tumor clusters in (I). **k**. Sequential t-SNE Clustering. The favorable survival t-SNE Wnt Cluster 1 tumors from Fig. 1i were re-analyzed using Notch Pathway transcripts, which generated the expected two t-SNE clusters (not show) with significant survival differences. (**l**). The unfavorable survival Cluster 2 tumors from Fig. 1i were similarly re-analyzed using Notch Pathway transcripts and were shown to be comprised of two sub-clusters with significant differences in long-term survival

Supplemental Fig. 1 summarizes our previous work [13] regarding the extent to which t-SNE-aided clustering of transcripts from 15 pathways with established roles in cancer [14, 16–18] can be used to predict long-term survival differences across all 34 cancer types representing 10,227 individual tumors from TCGA [9, 12, 13]. As an example, the analysis of 514 kidney clear cell carcinomas (KIRC) with the 23 transcripts comprising the Pyrimidine Biosynthesis Pathway generated two distinct t-SNE clusters containing nearly identical tumor numbers and associated with highly significant median survival differences (2090 days vs. > 4500 days, *P* = 5.6 × 10^− 7^, Fig. 1a and b and ref. [13]. A similar analysis performed on the same tumors with the 30 transcripts comprising the Notch Pathway also generated two distinct t-SNE clusters associated with significant survival differences (1912 days vs. 3554 days, *P* = 7.0 × 10^− 4^, Fig. 1c and d).

The fact that the above groups remained heterogeneous following t-SNE-based evaluation suggested that sequential analysis with transcripts from second pathway might further delineate the groups. We therefore re-analyzed tumors from the two Notch Pathway t-SNE clusters shown in Fig. 1c and d with transcripts from the Pyrimidine Biosynthesis Pathway. These results (Fig. 1e and f) showed that each Notch Pathway t-SNE cluster could be further divided into distinct Pyrimidine Biosynthesis Pathway t-SNE clusters. Specifically, the original favorable survival Notch Pathway Cluster 1 (median = 3554 days, Fig. 1d) was now shown to be comprised of an even more favorable group with median survival > 4500 days and a significantly more unfavorable group (median survival = 2386 days, *P* = 5.9 × 10^− 5^, Fig. 1e). This latter group was comparable in its survival to each of the short-term survival groups initially delineated with a single t-SNE analysis (*P* > 0.05 in each case). Similarly, analysis of the original unfavorable survival Notch Pathway Cluster 2 (median = 1912 days, Fig. 1d) also identified two clusters with significant survival differences (median = 3615 days vs. 1661 days, *P* = 0.023, Fig. 1f).

We next analyzed 511 low-grade gliomas using a similar sequential approach. Initial t-SNE profiling with transcripts from the Notch pathway identified two distinct Clusters with significant median long-term survival differences (3978 days vs. 1891 days, Fig. 1g and h, *P* = 3.0 × 10^− 4^). Analysis of the same tumors using the 25 transcripts from the Wnt Pathway produced four distinct t-SNE clusters (Fig. 1i). Of these, Cluster 1 individuals survived longer relative to Clusters 2 and 3 (median survival = 3200 days vs. 1915 days and 2433 days, respectively, *P* = 2.0 × 10^− 4^ in each case.

Clusters 1 and 2 each contained a sufficiently large tumor population to allow a meaningful second analysis to be performed with transcripts from the Notch Pathway. In the case of Wnt Cluster 1, this produced the expected two t-SNE Clusters similar to those seen in Fig. 1g (not shown) with significant differences in their median long-term survival (4695 days vs. 1933 days, *P* = 4.1 × 10^− 5^, Fig. 1k). A similar sequential analysis of the unfavorable Wnt Pathway Cluster 2 survival from Fig. 1i also produced two Notch Pathway t-SNE clusters with significantly different long-term survival of 4084 days and 1547 days (Fig. 1l, *P* = 0.01). A comparison of each of the favorable and unfavorable populations from Fig. 1k and l indicated significant differences in median survival (4695 days vs. 4084 days, *P* = 0.0034 and 1933 days vs. 1547 days, *P* = 0.008) as well as significant differences in survival when compared to most and least favorable survival obtained using only single t-SNE analyses (ex. 4695 days vs. 3978 days [Fig. 1h], P = 0.01 and 1547 days vs. 1891 days [Fig. 1h, *P* = 0.04]). Thus, unlike KIRCs, where a second t-SNE analysis was able to further subdivide groups into additional favorable or unfavorable long-term survival cohorts, neither of which survived significantly longer or shorter than those analyzed by only a single t-SNE analysis, the sequential t-SNE profiling of LGGs did identify patient subsets with particularly favorable or unfavorable survival that was well in excess of that predicted from the individual t-SNE analysis.

**Fig. 2:**
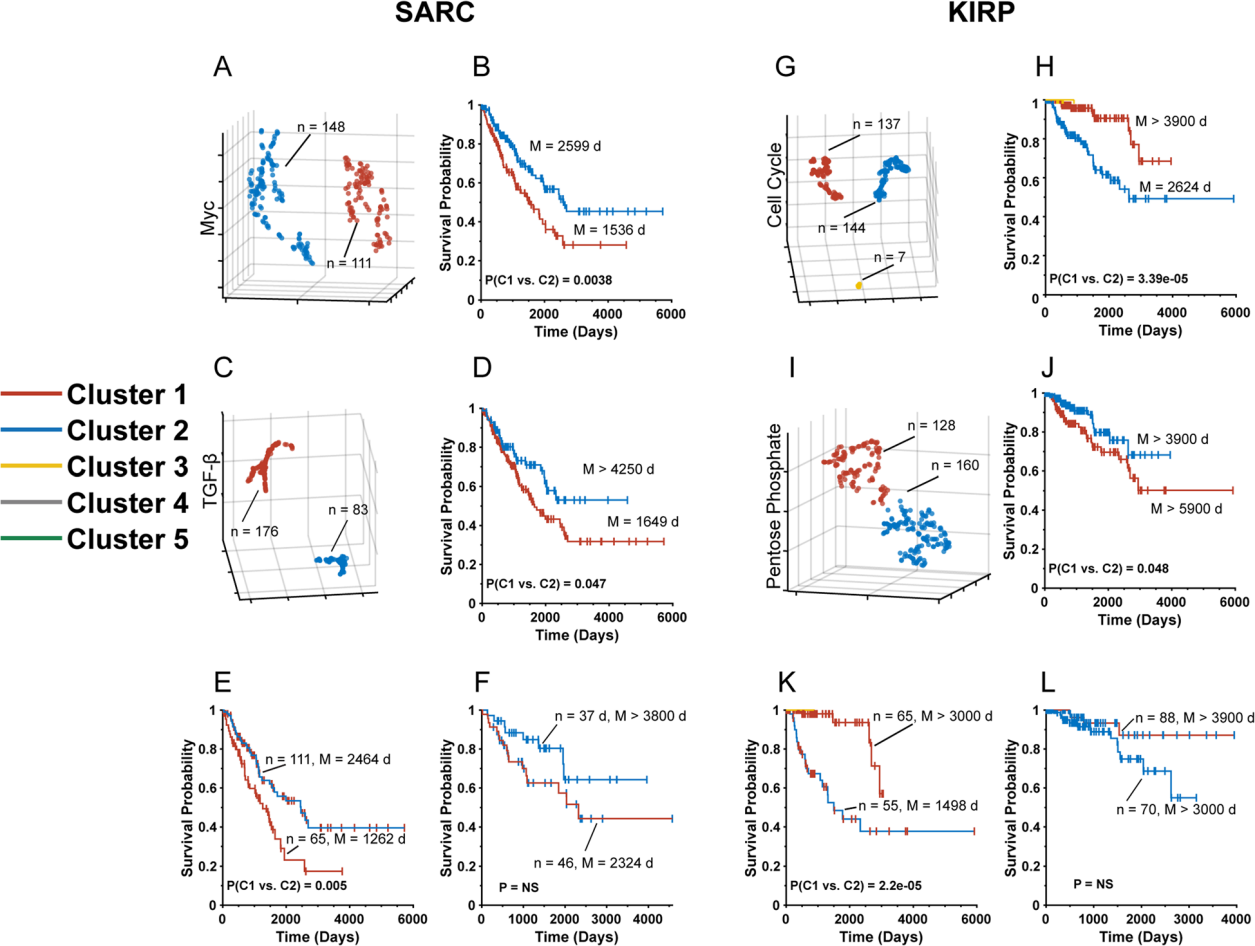
Sequential t-SNE analysis of SARC and KIRP. **a**. t-SNE-generated patterns of SARC Myc Pathway transcripts showing two distinct clusters. **b**. Kaplan-Meier survival curves of each of the tSNE clusters depicted in A. **c**. t-SNE analysis of SARC TGF-β Pathway transcripts. **d**. Kaplan-Meier survival curves of patients from each of the clusters shown in C. **e**. Kaplan-Meier curves of patients from the unfavorable survival TGF-β Pathway cluster from C after t-SNE analysis with Myc Pathway transcripts. Two t-SNE clusters similar to those depicted in A were generated (not shown). **F**. Kaplan-Meier survival of patients from the favorable survival TGF-β Pathway cluster shown in C after t-SNE analysis with Myc Pathway transcripts. Two t-SNE groups similar to those shown in A were generated (not shown). **g**. t-SNE analysis of KIRP Cell Cycle Pathway transcripts showing two major tumor clusters and a third comprised of only seven tumors. **h**. Kaplan-Meier survival curves of patients from Clusters 1 and 2 depicted in G. **i**. t-SNE-generated patterns of KIRP Pentose Phosphate Pathway transcripts showing two major clusters. **j** Kaplan-Meier survival curves of each of patients from the tumor clusters depicted in I. **k**. Kaplan-Meier curves of individuals from the unfavorable survival Pentose Phosphate Pathway cluster from I after t-SNE analysis using transcripts from the Cell Cycle Pathway. Two t-SNE clusters similar to those depicted in A were generated (not shown). **l**. Kaplan-Meier curves of individuals from the favorable survival Pentose Phosphate Pathway cluster from K after t-SNE analysis using transcripts from the Myc Pathway. Two t-SNE clusters similar to those depicted in A were generated (not shown)

To generalize these findings, we performed similar sequential t-SNE profiling on sarcomas (SARC) and kidney renal papillary cell carcinoma (KIRP) (Fig. 2). In the first case, 259 sarcomas were analyzed by t-SNE for differential expression patterns of transcripts comprising the Myc and TGF-β Pathways. Profiling of the Myc Pathway identified two t-SNE clusters with highly significant differences in median survival (1536 days [Cluster 1] vs. 2599 days [Cluster 2], *P* = 0.0038, Fig. 2a and b). Profiling of the TGF-β Pathway also identified two clusters with median survival of 1649 days (Cluster 1) and > 4500 days (Cluster 2), *P* = 0.047, Fig. 2c and d). Sequential t-SNE profiling of the TGF-β Pathway’s inferior survival cluster with Myc Pathway transcripts allowed it to be subdivided into two groups with median survival of 1262 days and 2464 days, *P* = 0.005, Fig. 2e). Similarly, the TGF-β Pathway t-SNE Cluster 2, comprising 83 individuals with superior median survival (> 4250 days, Fig. 2d), could also be divided into two groups. However, most likely because this group lacked a sufficiently large number of tumors, the two survival curves were not determined to be significantly different despite a clear trend in that direction (median survival 2324 days vs > 4500 days).

Analogously, t-SNE profiling of the 288 KIRPs using the 15 transcripts comprising the Cell Cycle Pathway [13] also generated two major clusters comprised of nearly identical numbers of tumors. A third t-SNE cluster comprised of only seven tumors was not analyzed further (Fig. 2g). Highly significant survival differences were observed between the first two groups (median survival> 3900 days [Cluster 1] vs. 2624 days [Cluster 2], *P* = 3.39 × 10^− 5^). t-SNE profiling of this same tumor population using the 11 transcripts comprising the Pentose Phosphate Pathway [13] also generated two distinct clusters (Fig. 2i) with borderline long-term survival differences (each > 3900 days, *P* = 0.048, Fig. 2j).

As before, significant improvements in survival prediction were achieved when the above tumor samples were subjected to sequential t-SNE analysis. Thus, when the inferior survival Pentose Phosphate Pathway Cluster 1 (Fig. 2i and j) was analyzed for the expression patterns of Cell Cycle Pathway transcripts, two t-SNE clusters with significantly different long-term median survival differences were obtained (1498 days vs. > 3000 days, *P* = 2.2 × 10^− 5^, Fig. 2k). The less favorable group’s 1498 day median survival time was significantly shorter than that of either of the less favorable groups from Cell Cycle Pathway and Pentose Phosphate Pathway t-SNE clusters [1498 days vs. 2624 days, *P* = 0.05 (Fig. 2h) and > 5900 days, *P* = 0.01 (Fig. 2j)]. Sequential t-SNE profiling on the favorable survival Pentose Phosphate Pathway Cluster 2 (Fig. 2i and j) with Cell Cycle Pathway transcripts did not demonstrate significant differences in the median survival times between the two resulting groups due most likely to sample number limitations and/or survival time constraints. Nevertheless, a clear trend was observed with 87% of the “favorable group” individuals (*n* = 88) remaining alive at ~ 3000 days versus only 55% of the “unfavorable group” individuals (*n* = 70) (Fig. 2l).

**Fig. 3:**
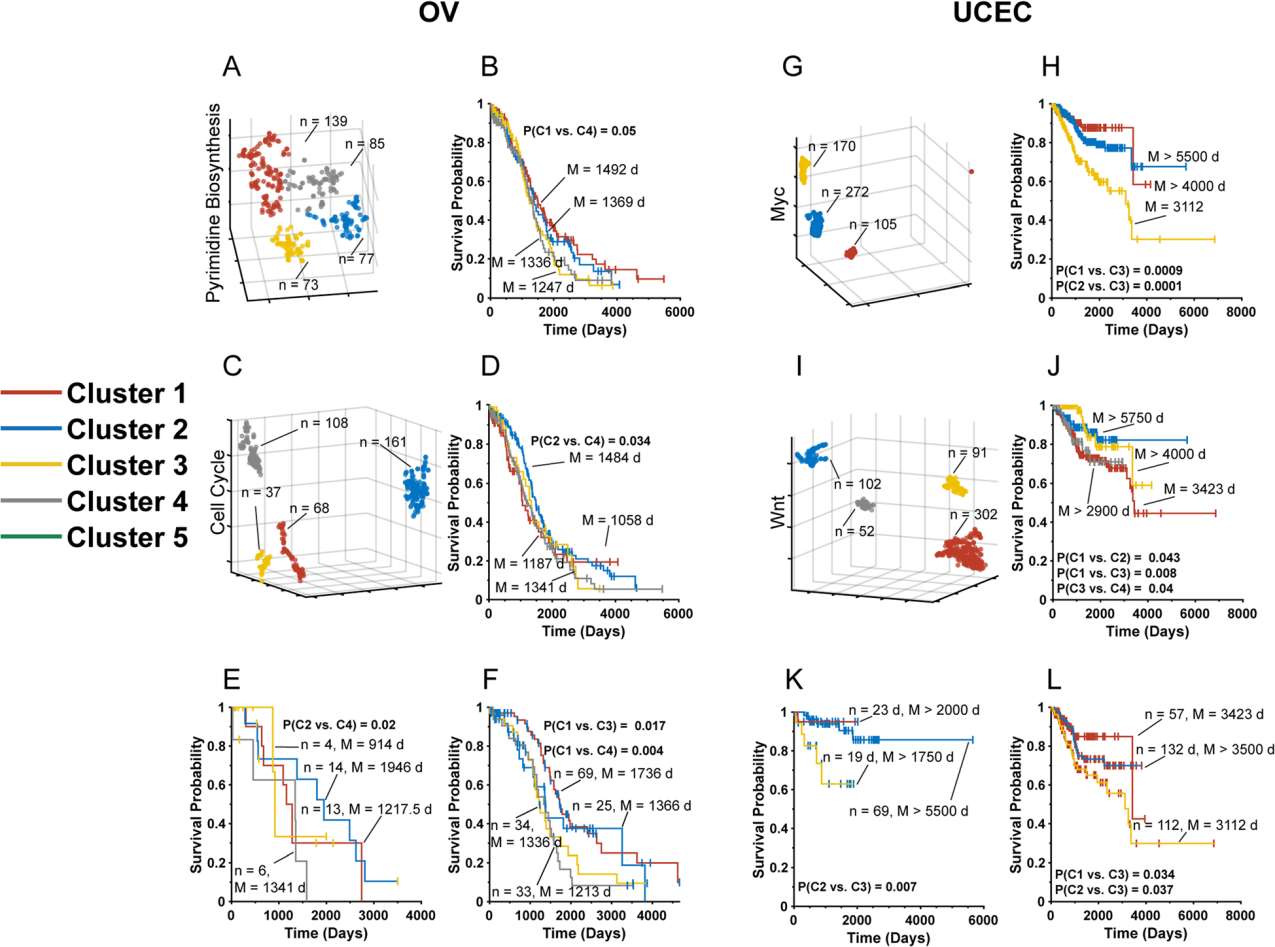
Sequential t-SNE analysis of OV and UCEC. **a**. t-SNE-generated patterns of Pyrimidine Biosynthesis Pathway transcripts showing four OV tumor clusters. **b**. Kaplan-Meier survival curves of patients from each of the Clusters depicted in A. **c**. t-SNE-generated patterns of OV Cell Cycle Pathway transcripts. **d**. Kaplan-Meier survival curves of patients from each of the Clusters shown in C. **e**. Kaplan-Meier survival curves of patients from unfavorable survival Cell Cycle Pathway Cluster 3 from C after t-SNE re-analysis using transcripts from the Pyrimidine Biosynthesis Pathway. Four t-SNE clusters similar to those depicted in A were generated (not shown) with two of these (Clusters 2 and 4) showing significant survival differences. **f**. Favorable survival Cell Cycle Pathway t-SNE Cluster 2 from (D) was analyzed with Pyrimidine Biosynthesis Pathway transcripts. Of these, Cluster 1 (median survival 1736 days) had a more favorable long-term survival than either Cluster 3 (1336 days) or Cluster 4 (1213 days) (*P* = 0.017 and *P* = 0.004, respectively. **g**. t-SNE-generated patterns of UCEC Myc Pathway transcripts. **h**. Kaplan-Meier survival of patients from the groups depicted in G. (I). t-SNE-generated patterns of UCEC Wnt Pathway transcripts. **j**. Kaplan-Meier survival curves of patients from each of the groups depicted in I. **k**. Kaplan-Meier survival curves of individuals from favorable survival Wnt Pathway t-SNE Cluster 2 following subsequent t-SNE profiling with Myc Pathway transcripts. **l**. Kaplan-Meier survival curves of patients from the unfavorable survival Wnt Pathway Cluster 1 cohort in K following repeat t-SNE profiling with Myc Pathway transcripts

Finally, we undertook a third analysis of ovarian (OV) and uterine corpus endometrial cancers (UCEC) whose t-SNE profiles were somewhat more complex and showed less pronounced inter-Cluster survival differences when interrogated with the transcripts of single pathways. For example, ovarian cancers generated four t-SNE clusters with Pyrimidine Biosynthesis Pathway transcripts (Fig. 3a). Of these, only Clusters 1 and 4 showed even borderline significant differences in their median long-term survival (1492 days vs. 1336 days, P = 0.05, Fig. 3b). Analysis of the same tumors using Cell Cycle Pathway transcripts also generated four distinct t-SNE clusters (Fig. 3c), with only Clusters 2 and 4 demonstrating modestly significant differences in median survival (1484 days vs. 1187 days, *P* = 0.034).

We sequentially profiled Cell Cycle Pathway Cluster 3 (median survival 1341 days, Fig. 3c) with Pyrimidine Biosynthesis Pathway transcripts. Due to the small size of the original Cell Cycle Pathway Cluster 3 (37 tumors) and the fact that its secondary analysis yielded four Pyrimidine Biosynthesis Pathway clusters, it was difficult to achieve a high degree of statistical significance among the four groups. Nevertheless, Clusters 2 and 4 showed significant differences in median survival (1946 days vs. 1341 days, respectively, *P* = 0.02, Fig. 3e). The much larger, 161 member Cell Cycle Cluster 2 (median survival 1484 days, Fig. 3d) could also be further sub-divided into four Pyrimidine Biosynthesis Pathway Clusters with significant median survival differences between some groups (Fig. 3f). For example, Cluster 1 (median survival 1736 days) showed significantly longer survival relative to both Cluster 3 (1336 days, *P* = 0.017) and Cluster 4 (1213 days, *P* = 0.004).

t-SNE profiling of Myc Pathway transcripts applied to 547 UCECs generated three distinct clusters (Fig. 3G) with Cluster 3 demonstrating a clear inferior median survival (3112 days) relative to the other two Clusters [each > 4000 days, *P* = 9.0 × 10^− 4^ (Cluster 1) and 1.0 × 10^− 4^ (Cluster 2), respectively]. Profiling with Wnt Pathway transcripts generated four clusters (Fig. 3i), with Cluster 1 having inferior median survival (3423 days) relative to Clusters 2 and 3 (> 3000 and > 3900 days, *P* = 0.043 and *P* = 0.008, respectively, Fig. 3j) and Cluster 3 showing a longer survival relative to Cluster 4 (P = 0.04).

Despite the favorable 82% long-term survival of Wnt Pathway Cluster 2 individuals (Fig. 3j), they could be further stratified into the expected three clusters following sequential analysis with Myc Pathway transcripts (not shown). Although the median survival of these clusters could not be determined, Cluster 3, which contained approximately one-firth of the individuals, showed significantly inferior survival relative to the other two Clusters (*P* = 0.007, Fig. 3k). Similarly, the subdivision of poor survival Wnt t-SNE Cluster 1 (Fig. 3j) using Myc Pathway transcripts identified one subgroup (Cluster 3, Fig. 3l) with particularly poor median survival (3112 days) relative to the other two Clusters (*P* = 0.034 and *P* = 0.037, respectively,).

Thus, in summary, the serial use of t-SNE to sub-classify expression patterns of transcripts from select cancer-related pathways made it possible to analyze multiple tumor types so as to achieve a higher degree of survival stratification than could be achieved with only a single t-SNE analysis. Thus, even after initial single Pathway analyses, tumor cohorts remained heterogeneous with regard to their cumulative long-term survival.

### Sequential hierarchical clustering/t-SNE profiling

Numerous studies have indicated that otherwise histologically similar tumors may nonetheless display distinct differences in their transcriptomes that correlate with survival and/or other behaviors [19–24]. We recently showed for some cancers that the ability to predict survival using this approach could be improved when combined with t-SNE profiling [13]. We decided to extend these findings by including a more comprehensive evaluation of all cancers in TCGA for which whole transcriptome profiling was available.

**Fig. 4:**
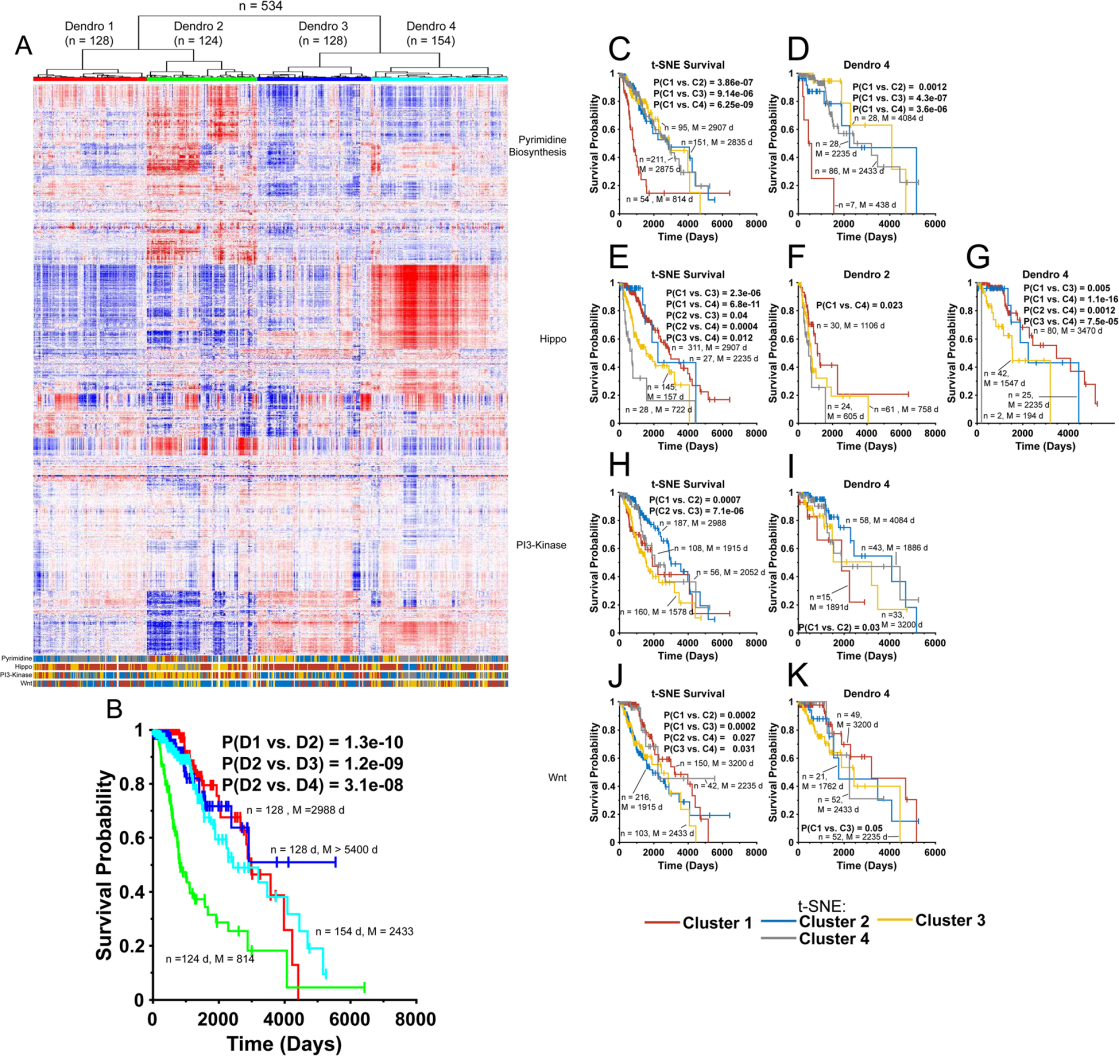
Sequential hierarchical clustering/t-SNE profiling of LGG. **a**. Hierarchical clustering of 534 LGG transcriptomes from TCGA showing four distinct groups (“Dendros’). At the bottom of the panel, the rows of colored bars represent the clusters into which each tumor was grouped following t-SNE analysis with that pathway’s transcripts. **b**. Kaplan-Meier survival of each Dendro (D1-D4) and the *P* values for each pair-wise comparison. **c**. Kaplan-Meier survival of all 534 LGGs based on the t-SNE Clusters to which they were assigned after profiling with Pyrimidine Biosynthesis Pathway transcripts. The number of tumors in each Cluster and the median survival are indicated as are the P values for significant pair-wise comparisons. **d**. Kaplan-Meier survival of patients from the 149 member Dendro 4 group based on their Pyrimidine Biosynthesis Pathway t-SNE Cluster identities. **e**. Kaplan-Meier survival for all LGG patients based on the t-SNE Clusters to which they were assigned after profiling with Hippo Pathway transcripts **f**. Kaplan-Meier survival of patients from the 115 Dendro 2 group based on their Hippo Pathway t-SNE Cluster identities. **g**. Kaplan-Meier survival of patients from the Dendro 4 group based on their Hippo Pathway t-SNE Cluster identities. **h**. Kaplan-Meier survival of all LGG patients based on the t-SNE Clusters to which they were assigned after profiling with PI3-kinase Pathway transcripts. **i**. Kaplan-Meier survival of patients from Dendro 4 based on their PI3-kinase Pathway t-SNE Cluster identities. **j**. Kaplan-Meier survival of all LGG based on the t-SNE Clusters to which they were assigned after profiling with Wnt Pathway Pathway transcripts. **k**. Kaplan-Meier survival of patients from Dendro 4 based on their Wnt Pathway t-SNE Cluster identities

Hierarchical clustering of the previously described LGG transcriptomes allowed the tumors to be divided into four groups [19], termed “Dendros 1–4” or “D1-D4” (Fig. 4a), with individuals in D2 having a particularly poor long-term survival relative to the others. (*P* < 3.1 × 10^− 8^) None of the remaining three Dendros showed any significant differences in survival (Fig. 4b).

Profiling the entire LGG group with 93 transcripts from four cancer-related pathways (Pyrimidine Biosynthesis, Hippo, PI3-kinase signaling and Wnt signaling) produced four t-SNE clusters in each case (not shown but see ref. [13]. When these Clusters were matched to the individual tumors in each of the Dendros, several non-random associations were seen. For example, t-SNE Cluster 1 of the Hippo Pathway contributed disproportionately to the Dendro 3 subset (*P* = 1.03 × 10^− 15^), whereas t-SNE Cluster 3 of the Hippo Pathway and t-SNE Cluster 3 of the PI3 kinase family of transcripts contributed disproportionately to the Dendro 2 group (*P* = 1.4 × 10^− 10^ and *P* = 2.95 × 10^− 7^, respectively) (Fig. 4a).

We next compared the survival of individuals in each t-SNE Cluster, either collectively or within the context of individual Dendro groups. In the first case, we found all tumors associated with Pyrimidine Biosynthesis Pathway t-SNE Cluster 1 to be associated with significantly shorter survival relative to the other t-SNE Clusters (*P* = 9.14 × 10^− 6^-6.25 × 10^− 9^, Fig. 4c). This was consistent with the disproportionate representation of these Cluster 1 tumors within Dendro 2 (*P* = 3.16 × 10^− 22^). Indeed, the only remaining Cluster 1 tumors were associated with Dendro 4 and while few in number (*n* = 7), the individuals in this group had a particularly short survival relative to those with tumors in the other t-SNE Clusters comprising this Dendro (*P* = 0.0012–4.3 × 10^− 7^, Fig. 4d).

Hippo Pathway Cluster 4 tumors also contributed disproportionately to Dendro 2 (*P* = 1.44 × 10^− 12^). Consistent with this, Cluster 4, both overall and in its Dendro 2 context, was associated with the shortest survival relative to the other t-SNE Clusters (*P* = 0.023–6.8 × 10^− 11^, Fig. 2e&f). The only remaining Hippo Pathway Cluster 4 tumors were associated with Dendro 4. While associated with extremely short survival, they were too few in number (*n* = 2) to make a reliable statement concerning the significance of this. However, individuals with tumors in Dendro 4 (median survival = 2433 d) could be further distinguished by a long-term survival t-SNE 1 Cluster (median survival = 3470 d) and a shorter-term survival t-SNE 3 Cluster (median survival = 1547 d) (Fig. 4g).

Similar associations could be made in the case of PI3-kinase Pathway transcripts where, across all tumors t-SNE Cluster 2 individuals had longer survival than either Cluster 1 or Cluster 3 individuals (*P* = 7.0 × 10^− 4^ and.

7.1 × 10^− 6^, respectively, Fig. 4h). Additionally, t-SNE Clusters 1 and 2 clearly could be used to further delineate survival within the Dendro 4 cohort (median survival =1891 d vs. 3200 d, respectively, *P* = 0.03, Fig. 4i).

Finally, the four t-SNE Clusters generated from Wnt Signaling Pathway transcripts were associated with significant differences in survival across all tumors (Fig. 4j). Among the most significant of these were the inferior survival of individuals with tumors in Cluster 1 vs. Cluster 2 and Cluster 1 vs. Cluster 3 (*P* = 2.0 × 10^− 4^ in each case). Furthermore, the survival difference between Clusters 1 and 3 could be utilized in an analysis of the Dendro 4 cohort to improve overall survival prediction within this group (median survival 3200 d vs. 2235 d, *P* = 0.05, Fig. 4k).

**Fig. 5:**
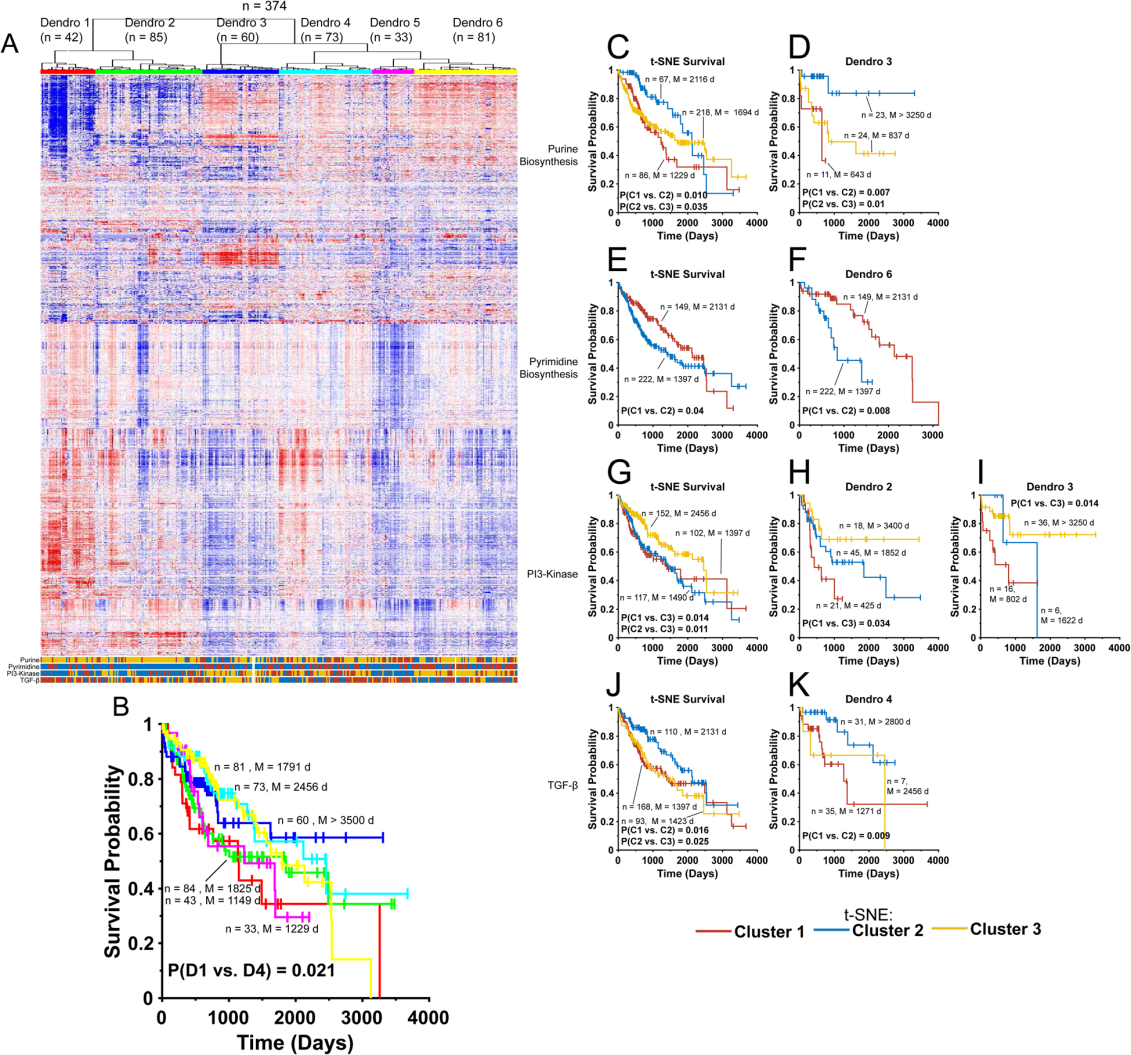
Sequential hierarchical clustering/t-SNE profiling of HCC. **a**. Hierarchical clustering of 374 HCC transcriptomes from TCGA showing six Dendros. At the bottom of the panel, the colored bars represent the results of t-SNE profiling performed with the four indicated transcript pathways. Each HCC was assigned a t-SNE Cluster identity within the indicated family as described in the legend to Fig. 4. **b**. Kaplan-Meier survival of patients from each Dendro (D1-D6). The only significant difference among the six groups was D1 vs. D4 (*P* = 0.021). **c**. Kaplan-Meier survival of all 374 HCC patients based on the three Clusters generated by t-SNE profiling of tumors with Purine Biosynthesis Pathway transcripts. The number of tumors in each Cluster and the median survival are indicated as are the P values for significant pair-wise comparisons. **d**. Kaplan-Meier survival of the 58 patients from Dendro 3 based on the Purine Biosynthesis Pathway t-SNE Cluster identities of their tumors. **e**. Kaplan-Meier survival for all patients based on the two Clusters generated by t-SNE profiling of tumors with Pyrimidine Biosynthesis Pathway transcripts. **f**. Kaplan-Meier survival of patients from Dendro 6 based on the two Pyrimidine Biosynthesis Pathway t-SNE Cluster identities of their tumors. **g**. Kaplan-Meier survival of all patients based on the three t-SNE Clusters to which their tumors were assigned after profiling with PI-3 Kinase Pathway transcripts. **h**. Kaplan-Meier survival of patients from the Dendro 2 group based on the three PI3-Kinase Pathway t-SNE Clusters to which their tumors were assigned. **i**. Kaplan-Meier survival of patients from the Dendro 3 group based on the three PI3-Kinase Pathway t-SNE Clusters to which their tumors were assigned. **j**. Kaplan-Meier survival for all patients based on the three TGF-β Pathway transcript t-SNE Clusters to which their tumors were assigned. **k**. Kaplan-Meier survival of patients from Dendro 4 based on the three TGF-β Pathway t-SNE Clusters to which their tumors were assigned. Small discrepancies in numbers of patients are due to slight differences in which patients were hierarchically clustered, and/or to missing or incomplete survival data

Another example in which the tandem sequential hierarchical clustering/t-SNE approach was found to be particularly useful in allowing more refined stratification of patient survival was seen in the case of 374 hepatocellular carcinomas (HCCs). For these tumors, hierarchical clustering generated six Dendros which showed only relatively modest survival differences (Dendro 1 vs. Dendro 4, *P* = 0.021, Fig. 5a and b). t-SNE profiling with four pathways (Purine Biosynthesis, Pyrimidine Biosynthesis, PI3-kinase signaling and TGF-β signaling), performed either alone or sequentially on each Dendro was far more useful in identifying subsets of patients with particularly favorable or unfavorable long-term survival. For example, t-SNE profiling alone of all tumors with Purine Biosynthesis Pathway transcripts identified three Clusters with significant differences between Clusters 1 and 2 (median survival = 1229 d vs. 2116 d, respectively (*P* = 0.01 and ref. [13] and Clusters 2 and 3 (median survival = 2116 days vs. 1694 days, respectively, *P* = 0.035) (Fig. 5c). When t-SNE profiling with Purine Biosynthesis Pathway transcripts was applied to Dendro 3 however, much more substantive differences in survival were observed, with Clusters 1 and 2 showing median survivals of 643 days and > 3500 days (*P* = 0.007) and Clusters 2 and 3 demonstrating median survivals of > 3500 days and 837 days (P = 0.01) (Fig. 5d).

t-SNE profiling of all HCCs with transcripts from the Pyrimidine Biosynthesis Pathway generated two Clusters with significant survival differences (2131 days vs. 1397 days, *P* = 0.04, Fig. 5e). However, the 734 day difference in these median survivals was significantly extended to 1283 days when the Dendro 6 cohort of patients was divided according to t-SNE cluster, where median survivals of 2131 days and 848 days were obtained (*P* = 0.008) (Fig. 5f).

Additional t-SNE profiling of PI3-kinase Pathway signaling transcripts was also found to be useful when used to evaluate all HCCs. Three clusters were identified with significant survival differences between Clusters 1 and 3 (1397 days vs. 2456 days, *P* = 0.014) and between Clusters 2 and 3 (1490 days vs. 2456 days, *P* = 0.011) being observed (Fig. 5g). As before, increased survival stratification was achieved when t-SNE profiling was applied against Dendro 2 where Clusters 1 and 3 showed median survival differences of 425 days vs. > 3500 days (*P* = 0.034) (Fig. 5h). When applied against Dendro 3, Clusters 1 and 3 showed similarly large disparities in median survival (802 days vs. > 3500 days, respectively, P = 0.034) (Fig. 5i).

Lastly, the three t-SNE Clusters of TGF-β Pathway transcripts were associated with differential survival among all individuals with HCC (Fig. J and ref. [13]). Significant differences in median survival were observed for Clusters 1 vs 2 (1397 days and 2131 days, respectively, *P* = 0.016) and for Clusters 2 and 3 (2131 days vs. 1423 days, *P* = 0.025). However, when applied only to the Dendro 4 group, t-SNE profiling of TGF-β Pathway transcripts was able to discern highly significant survival differences between Clusters 1 and 2 (median survival = 1271 days vs. > 3500 days, *P* = 0.009 (Fig. 5k).

### A comprehensive, interactive collection of human cancers amenable to sequential analysis

Given the ability of sequential profiling to improve survival stratification, we constructed an interactive website (https://chpupsom19.shinyapps.io/Survival_Analysis_tsne_umap_TCGA and https://github.com/RavulaPitt/Sequential-t-SNE/). that allows the transcriptional profiles of > 10,000 specimens from 34 different human cancers in TCGA to be queried using either of the approaches described above. In addition to the limited number of examples shown here (Figs. 1, 2, and 3), this website allows for the sequential t-SNE analysis of all tumor groups in TCGA using any of the pathways that revealed survival differences among t-SNE clusters (Suppl. Fig. 1 and ref. [13]). An additional section of the website permits tumors whose whole transcriptome profiles correlate with survival differences to be secondarily analyzed by t-SNE (Figs. 4 and 5). This is particularly useful for some of the larger TCGA cancer cohorts such as KIRC, breast cancer and non-small cell lung cancer, where well over 500 well-curated samples in each group are available. Factors other than the total sample size, which that can limit the robustness of these types of analyses, include the number of Dendros and t-SNE Clusters.

## Discussion

Biological and clinical heterogeneity among otherwise histologically indistinguishable tumors explains the vast majority of therapeutic failures and provides the major rationale for individualizing, or “personalizing”, cancer treatment. Thus far, the means of attaining such precision medicine-based goals has involved a combination of improved clinical staging; high-resolution imaging techniques; immuno-histochemical-based tumor sub-classification and, increasingly, molecular and pharmacogenomic evaluation to stratify individuals according to inherent risk and likelihood of response to chemotherapeutic regimens [5, 25–38]. The deployment of newer techniques such as liquid biopsies, which quantify circulating tumor DNA, promise to provide additional benefits by allowing serial assessments of response to therapy or the detection of impending recurrence in cases where the tumor has been previously resected or otherwise rendered undetectable by standard methods [39–42]. Recently a robust dynamic model that allows for the integration of a variety of diverse outcome predictors acquired over time into a single profile and dubbed “Continuous Individualized Risk Index” (CIRI) has been described for patients with breast cancer, chronic lymphocytic leukemia and diffuse large B cell lymphoma. This makes possible ongoing evaluation using a combination of pre-treatment risk factors, interim risk factors obtained shortly after the initiation of therapy and end of treatment risk factors [43]. It seems reasonable to presume that this type of combinatorial evaluation might provide most advantageous in those patients whose initial pre-treatment molecular profiles such as those described here are best able to first classify them into high or low risk groups.

Collectively, the findings of this report confirm and significantly expand upon our previous results in a small number of cancer types, which demonstrated that the survival heterogeneity that remains after sub-classification of tumors using either a single round of t-SNE or whole transcriptome profiling can be further minimized by a second t-SNE-based analysis. In some cases, this allows for significant improvements in survival stratification beyond those achievable using only a single analysis [13]. Such information, obtained at the time of diagnosis offers the potential for better patient stratification into various risk groups thereby allowing for more precise and appropriate therapeutic choices and decisions regarding the nature of long-term follow up in much the same way as is now used for more standard, molecular-based assessments [1, 3–8].

One limitation of this sequential method is the inability to apply t-SNE profiling in four of the tumor types contained within the TCGA data base [13]. These include diffuse large B-cell lymphomas, squamous cell lung cancers, phenochromocytomas/ paraganglioneuromas and testicular germ cell tumors (Supplemental Fig. 1). However, we have thus far applied t-SNE analysis to only 15 pathways comprised of 362 transcripts. It seems likely that, as other pathways are added, they will prove useful for the evaluation of tumors whose analyses by this method have remained elusive.

A second and more important limitation of our approach arises as a result of relatively small numbers of tumors in the TCGA population, making it difficult to maintain statistically robust survival differences among groups as they are progressively subdivided during the course of sequential analysis. An example of this was encountered in the case of HCCs where, despite an initial group of 377 samples, relatively small subsets, each comprised of 42–85 tumors, were obtained after hierarchical clustering into six Dendros (Fig. 5A). Subsequent subdivision of these individual groups into as many as four t-SNE clusters (Fig. 5G and H) further reduced the number of evaluable samples and in some cases, made statistically valid survival distinctions among groups more uncertain if not impossible despite clear trends indicating otherwise. In contrast, instances in which both initial and sequential evaluation identified only small numbers of cohorts and/or contained more tumor samples, often provided more robust survival outcomes. Thus, the initial t-SNE-based evaluation of 514 KIRCs with transcripts from the Pyrimidine Biosynthesis identified only two clusters for subsequent analysis with Notch Pathway transcripts which also yielded only two t-SNE clusters for a total of four groups for which survival differences could be computed (Fig. 1a-f). The ability to obtain such high-quality survival information from currently available data sets, such as those from TCGA, is likely to increase as RNAseq is more routinely utilized and the content of existing data bases expands. The fact that many tumors can be evaluated by t-SNE with transcripts from multiple pathways can also be utilized advantageously by empirically evaluating those cases in which the number of t-SNE clusters or hierarchically clustered Dendros is minimized and/or which identify the most significant differences in long-term survival. In this regard, it is notable that 19 of the 34 cancer types in TCGA can be stratified for survival based the t-SNE profiles of transcripts from at least three of the 15 pathways (Supplementary Fig. 1). This, combined with the increasingly large number of samples available for analysis, may also eventually allow for more than two sequential analysis to be employed.

It is important to reiterate why the relative expression levels (i.e. the patterns) of small groups of functionally related transcripts likely serve as powerful predictors of long-term survival in much the same manner, and in some cases better, as whole transcriptome profiling [19–21, 44, 45]. The steady-state gene expression levels of these various groups represent the integration of the differential activities, sequence-specific and epigenetically determined binding affinities of various transcription factors that regulate these genes; the composition and activities of multi-component proximal promoter- and enhancer-binding general transcription complexes such as RNA polymerase II and Mediator and the overall chromatin landscape that restricts the access of these factors to their target DNA regions [46–52]. Collectively, RNA steady state levels are additionally influenced by various post-transcriptional modifications such as the efficiency of mRNA splicing, secondary structure and base modification [53–55]. These patterns therefore represent surrogate reporters for the unique transcriptional environments that distinguish the various molecular subclasses of most cancers and their attendant behaviors. The likelihood that the regulation of transcripts representing a specific, functionally-related family differs from that other families and from the more general regulation of the entire transcriptome may explain why the sequential approach described can be utilized with success.

## Conclusions

The stratification of cancer patients into favorable or unfavorable prognostic groups at the time of diagnosis is essential to choosing the most appropriate therapeutic options and long-term monitoring protocols. Molecular analysis, generally based on whole transcriptome expression profiles of tumors, has played an increasingly important role in informing these clinical decisions. However, even when classified in this manner, significant heterogeneity often remains within the individual tumor subsets. The work presented here indicates that our previously described t-SNE-based method of long-term survival prediction that relies on the patterns of expression of small numbers of transcripts derived from 15 cancer cell signaling, proliferation and metabolic pathways [9, 12, 13] can be significantly enhanced when two pathways’ transcripts are analyzed sequentially by t-SNE or when t-SNE profiling is applied to tumors that have first been stratified by whole transcriptome profiling. This tandem approach holds the promise of becoming more robust and reliable as both tumor data bases and the number of pathways employed expand. Most importantly, the identification of distinct patterns of expression in the cancer-related pathways described here at the time of diagnosis that are associated with distinct differences in long-term survival offers the potential to assist clinicians with therapeutic and long-term follow-up decisions.

## Supplementary information

**Additional file 1: Supplemental Fig. S1** Summary of the predictive value of t-SNE-assisted clustering of functionally-related transcripts. Each column indicates the pathway whose component transcripts were used to generate t-SNE profiles of the 34 TCGA cancers indicated along the left border. The number of transcripts comprising each pathway are indicated in parentheses at the tope of each column. See refs. [9, 12, 13], and for the identities of the individual transcripts comprising these pathways. Colored boxes show the tumor groups for which the indicated pathway’s transcripts generated multiple t-SNE cluster, at least two of which showed significant differences in long-term survival based on Kaplan-Meier analysis. The color of each box indicates the *P* value for the most disparate survival differences as shown by the key at the right. At the bottom of each column is shown the number of tumor types, the total number of tumors and the per cent of all tumors for which the indicated pathway was informative for long-term survival. The total number of colored boxes across each row indicates the number of pathways that were capable of identifying t-SNE clusters with significant survival differences for that tumor type. Grey boxes indicate those groups in which inter-cluster survival differences were not significant or in which only a single cluster was generated by t-SNE profiling.

## Data Availability

The work described here is partly based on data generated from TCGA Research Network (http://cancergenome.nih.gov). Clinical annotation files and transcriptomic data were downloaded from UCSC Xenabrowser (https://xenabrowser.net) (University of CA). The software used for tSNE profiling is available at https://projector.tensorflow.org/).

## Abbreviations

t-SNE: T-Distributed stochastic nearest neighbor embedding
TCGA: The Cancer Genome ATLAS
