# Supplementary material for: Sequential analysis of transcript expression patterns improves survival prediction in multiple cancers
Source: BMC Cancer. 2020 Apr 7;20:297. doi: 10.1186/s12885-020-06756-x (PMC7140376; doi:10.1186/s12885-020-06756-x)
Supplement: Supplementary file 1 — Additional file 1: Supplemental Fig. S1 Summary of the predictive value of t-SNE-assisted clustering of functionally-related transcripts. Each column indicates the pathway whose component transcripts were used to generate t-SNE profiles of the 34 TCGA cancers indicated along the left border. The number of transcripts comprising each pathway are indicated in parentheses at the tope of each column. See refs. [9, 12, 13], and for the identities of the individual transcripts comprising these pathways. Colored boxes show the tumor groups for which the indicated pathway’s transcripts generated multiple t-SNE cluster, at least two of which showed significant differences in long-term survival based on Kaplan-Meier analysis. The color of each box indicates the P value for the most disparate survival differences as shown by the key at the right. At the bottom of each column is shown the number of tumor types, the total number of tumors and the per cent of all tumors for which the indicated pathway was informative for long-term survival. The total number of colored boxes across each row indicates the number of pathways that were capable of identifying t-SNE clusters with significant survival differences for that tumor type. Grey boxes indicate those groups in which inter-cluster survival differences were not significant or in which only a single cluster was generated by t-SNE profiling. [file 12885_2020_6756_MOESM1_ESM.pdf]

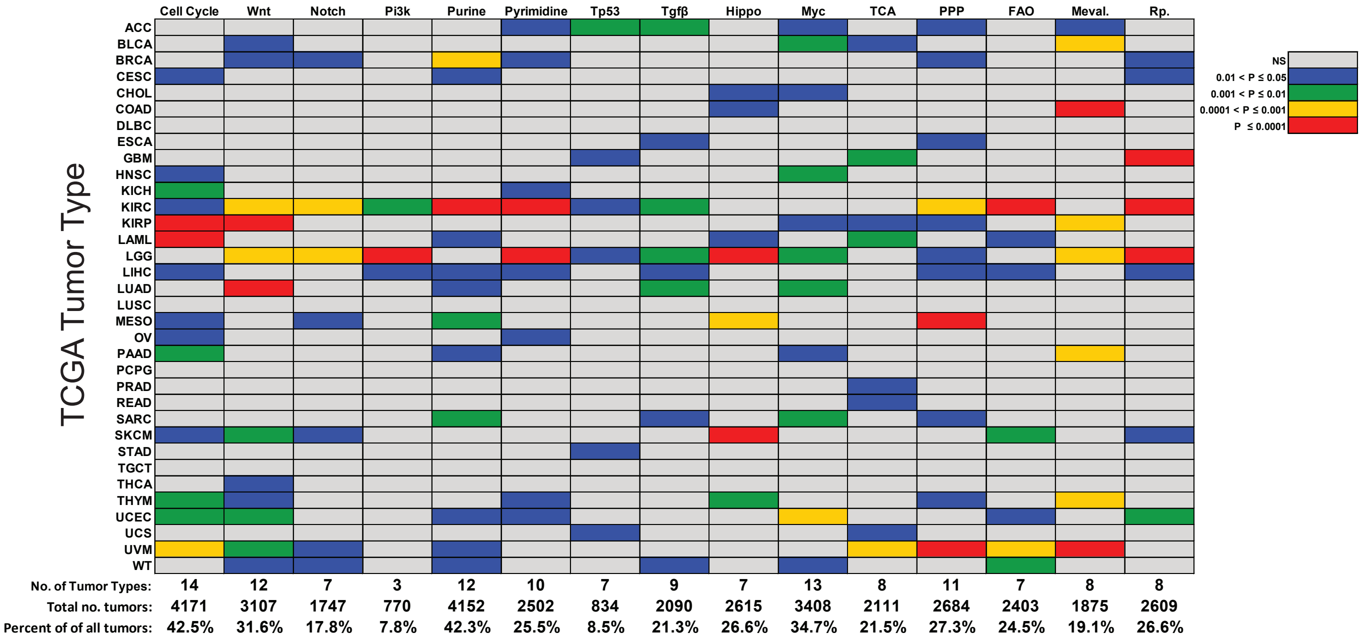
TCGA Tumor Type
Cell Cycle
Wnt
Notch
Pi3k
Purine
Pyrimidine
Tp53
Tgfβ
Hippo
Myc
TCA
PPP
FAO
Meval.
Rp.
ACC
BLCA
BRCA
CESC
CHOL
COAD
DLBC
ESCA
GBM
HNSC
KICH
KIRC
KIRP
LAML
LGG
LIHC
LUAD
LUSC
MESO
OV
PAAD
PCPG
PRAD
READ
SARC
SKCM
STAD
TGCT
THCA
THYM
UCEC
UCS
UVM
WT
NS
0.01 < P ≤ 0.05
0.001 < P ≤ 0.01
0.0001 < P ≤ 0.001
P ≤ 0.0001
No. of Tumor Types: 14 12 7 3 12 10 7 9 7 13 8 11 7 8 8
Total no. tumors: 4171 3107 1747 770 4152 2502 834 2090 2615 3408 2111 2684 2403 1875 2609
Percent of of all tumors: 42.5% 31.6% 17.8% 7.8% 42.3% 25.5% 8.5% 21.3% 26.6% 34.7% 21.5% 27.3% 24.5% 19.1% 26.6%
